# Editorial: Complexity of tumor microenvironment: A major culprit in cancer development, volume II

**DOI:** 10.3389/fendo.2022.1126778

**Published:** 2023-01-12

**Authors:** Ihtisham Bukhari, Yuanwei Zhang, Rick Francis Thorne, Yang Mi

**Affiliations:** ^1^Henan Key Laboratory of Helicobacter pylori, Microbiota and Gastrointestinal Cancers, Marshall Medical Research Center, Fifth Affiliated Hospital of Zhengzhou University, Zhengzhou, China; ^2^Translational Research Institute, Henan Provincial and Zhengzhou City Key Laboratory of Non-coding RNA and Cancer Metabolism, Henan International Join Laboratory of Non-coding RNA and Metabolism in Cancer, Henan Provincial People’s Hospital, Academy of Medical Sciences, Zhengzhou University, Zhengzhou, Henan, China; ^3^School of Life Sciences, University of Science and Technology of China, Hefei, Anhui, China; ^4^School of Environmental and Life Sciences, University of Newcastle, Callaghan, NSW, Australia

**Keywords:** tumor microenvironment, immunotherapy, cancer management, survival and prognosis, cancer biomarkers

The tumor microenvironment (TME) consists of tumor cells along with various immune, stromal, and endothelial cells, along with extracellular matrix components (1–3). Notably, complex interactions and material exchanges between the tumor microenvironment and its surroundings support tumor growth and progression (4, 5). Thus, a proper understanding of the TME requires a thorough study of its components and interactions (5, 6) with such information being essential for defining the underlying mechanisms of cancer development and progressing anti-cancer therapies for routine clinical use (7–10).

Various factors have been associated with cancer development and progression including recent research highlights concerning the metabolic (11–13) and other molecular factors (14–16). Notably, it has been well-established that metabolism and infiltrating cells in the TME represent attractive prognostic markers across a range of cancer types (17, 18). Additionally, recent work has also better delineated the role of lncRNAs and miRNAs, particularly their essential contribution in the TME as mediators of cancer progression and drug resistance (19–22). Indeed, such information has helped popularize the notion of using RNA-based targeting in future cancer management strategies (23–25).

Other recent advances in cancer therapeutic approaches have shifted the focus back toward the immunotherapy (26, 27). Surprisingly, the routine clinical introduction of cell-based immunotherapy has completely resketched the treatment landscape of the cancer management (28–30). Genetically modified immune cells such as CAR-T cells have produced incredible responses in some solid tumors (31–33). These immune cells have enhanced functional ability and protection from negative signals from immune checkpoints and the hostile tumor microenvironment (34). However, more therapeutic precision is still required to target tumors accurately. Moreover, multiplexed precision genome editing with pros and cons has become a highly flexible and modular toolkit for specifically addressing the challenges of precision interventions (35–37). Towards better functionality, genome editing tools like CRISPR/Cas9 and TALENs have made it possible to create additional genetic modifications in immune cells (36, 38, 39). Such methods have also been modified for better efficiency and safety in the therapeutic applications (40, 41) although off-target and other hidden effects of genome editing are major concerns for their widespread routine use in clinics. Thus, more comprehensive studies related to the genetic modifications of immune and cancer cells are required to gain sufficient confidence for application as standard cancer therapy.

For the current research topic, we aimed to collect research studies on the advancements in the tumor microenvironment and its roles in cancer regulation and clinical applications. After an extensive selection and peer review, only four articles were selected exploring new dimensions in this research field.

Firstly, Catellani et al. reviewed the role of lncRNAs and miRNAs in regulating growth hormones and insulin-like growth factor, both of which are crucial for biological processes including cellular proliferation, differentiation, and angiogenesis of various cancers including pituitary adenomas, osteosarcomas, and colorectal cancer. Wu et al. studied the clinical and biological importance of glucocorticoid receptors (GR) in adrenocortical carcinoma (ACC) patients. Their extensive analyses showed that GR positively correlates with higher cortisol levels, immune function, and poorer survival in ACC patients. This work advances GR as a potential ACC biomarker for use in prognostic and therapeutic settings although further work is required to obtain enough confidence before being used clinically. Ruan et al., analyzed a cohort of 5221 mixed cancer patients to define the relationship between inflammation and insulin resistance markers. Among the studied markers, the CRP-TyG index (CTI) was revealed as an indicator of poor prognosis that could be considered for treatment stratifications in multiple cancer types. Lastly, it is well documented that the E3-ubiquitin ligase TRIM21 (Tripartite Motif Containing-21) acts in dual capacities as either a tumor suppressor or promotor, altering metabolic and inflammatory pathways in different cancers. To shed further light on TRIM21, Chen et al. reviewed the participation of TRIM21 in cancer development, cancer-associated immune responses and potential roles in cancer immunity and therapeutics. Considering that TRIM21 appears to display dual roles in cancer development, some caution is required if this enzyme is to be considered as a therapeutic target.

## Conclusion and prospects

Although thousands of potential cancer biomarkers have been suggested, very few are currently used confidently in clinics for diagnostic, prognostic, and therapeutic applications. The role of the TME in cancer progression is being increasingly appreciated and therefore further focus is required to better understand its unique cellular and extracellular composition and function. In turn, more wholistic knowledge of the TME is expected to help translate basic research into more effective outcomes, rewriting clinical guidelines to include new biomarkers, therapeutic targets and treatment approaches.

## Author contributions

All authors listed have made a substantial, direct, and intellectual contribution to the work and approved it for publication.
